# Ruling out Pulmonary Embolism in Patients with (Suspected) COVID-19—A Prospective Cohort Study

**DOI:** 10.1055/s-0041-1735155

**Published:** 2021-09-15

**Authors:** Milou A.M. Stals, Fleur H.J. Kaptein, Remy H.H. Bemelmans, Thomas van Bemmel, Inge C. Boukema, Dionne C.W. Braeken, Sander J.E. Braken, Carlinda Bresser, Hugo ten Cate, Duco D. Deenstra, Yordi P.A. van Dooren, Laura M. Faber, Marco J.J.H. Grootenboers, Lianne R. de Haan, Carolien Haazer, Antonio Iglesias del Sol, Sarah Kelliher, Ted Koster, Lucia J.M. Kroft, Rick I. Meijer, Fleur Pals, Eric R.E. van Thiel, Peter E. Westerweel, Marije ten Wolde, Frederikus A. Klok, Menno V. Huisman, on behalf of the Dutch COVID & Thrombosis Coalition (DCTC)

**Affiliations:** 1 Department of Thrombosis and Hemostasis, Leiden University Medical Center, Leiden, South-Holland, The Netherlands; 2 Department of Internal Medicine, Hospital Gelderse Vallei, Ede, Gelderland, The Netherlands; 3 Department of Internal Medicine, Gelre Ziekenhuizen Apeldoorn, Apeldoorn, Gelderland, The Netherlands; 4 Department of Internal Medicine, Amsterdam UMC Locatie VUmc, Amsterdam, Noord-Holland, The Netherlands; 5 Thrombosis Expertise Center, Maastricht University Medical Center, Maastricht, Limburg, the Netherlands; 6 Department of Hematology, Red Cross Hospital, Beverwijk, Noord-Holland, The Netherlands; 7 Thrombosis Expertise Center, Maastricht University Medical Centre + , Maastricht, Limburg, The Netherlands; 8 Department of Pulmonology, Catharina Hospital, Eindhoven, North Brabant, The Netherlands; 9 Department of Pulmonology, Groene Hart Hospital, Gouda, Zuid-Holland, The Netherlands; 10 Department of Hematology, Red Cross Hospital, Beverwijk, Noord-Holland, The Netherlands; 11 Department of Pulmonology, Amphia Hospital, Breda, North Brabant, The Netherlands; 12 Department of Internal Medicine, Flevo Hospital, Almere, Flevoland, The Netherlands; 13 Department of Internal Medicine, Reinier de Graaf Hospital, Delft, Zuid-Holland, The Netherlands; 14 Department of Internal Medicine, Alrijne Hospital Location Leiderdorp, Leiderdorp, Zuid-Holland, The Netherlands; 15 Department of Hematology, Mater Misericordiae University Hospital, Dublin, Ireland; 16 Department of Internal Medicine, Groene Hart Hospital, Gouda, Zuid-Holland, The Netherlands; 17 Department of Radiology, Leiden University Medical Center, Leiden, Zuid-Holland, The Netherlands; 18 Department of Internal Medicine Amsterdam UMC Locatie VUMC, Amsterdam, Noord-Holland, The Netherlands; 19 Department of Pulmonology, Albert Schweitzer Hospital, Dordrecht, Zuid-Holland, The Netherlands; 20 Department of Internal Medicine, Albert Schweitzer Hospital, Dordrecht, Zuid-Holland, The Netherlands

**Keywords:** venous thromboembolism, diagnosis, pulmonary embolism, COVID-19

## Abstract

**Background** Diagnostic strategies for suspected pulmonary embolism (PE) have not been prospectively evaluated in COVID-19 patients.

**Methods** Prospective, multicenter, outcome study in 707 patients with both (suspected) COVID-19 and suspected PE in 14 hospitals. Patients on chronic anticoagulant therapy were excluded. Informed consent was obtained by opt-out approach. Patients were managed by validated diagnostic strategies for suspected PE. We evaluated the safety (3-month failure rate) and efficiency (number of computed tomography pulmonary angiographies [CTPAs] avoided) of the applied strategies.

**Results** Overall PE prevalence was 28%. YEARS was applied in 36%, Wells rule in 4.2%, and “CTPA only” in 52%; 7.4% was not tested because of hemodynamic or respiratory instability. Within YEARS, PE was considered excluded without CTPA in 29%, of which one patient developed nonfatal PE during follow-up (failure rate 1.4%, 95% CI 0.04–7.8). One-hundred seventeen patients (46%) managed according to YEARS had a negative CTPA, of whom 10 were diagnosed with nonfatal venous thromboembolism (VTE) during follow-up (failure rate 8.8%, 95% CI 4.3–16). In patients managed by CTPA only, 66% had an initial negative CTPA, of whom eight patients were diagnosed with a nonfatal VTE during follow-up (failure rate 3.6%, 95% CI 1.6–7.0).

**Conclusion** Our results underline the applicability of YEARS in (suspected) COVID-19 patients with suspected PE. CTPA could be avoided in 29% of patients managed by YEARS, with a low failure rate. The failure rate after a negative CTPA, used as a sole test or within YEARS, was non-negligible and reflects the high thrombotic risk in these patients, warranting ongoing vigilance.

## Take Home Message

In our study, CTPA could be avoided in 29% of patients managed by YEARS with a low failure rate, underlining the applicability of the YEARS algorithm in (suspected) COVID-19. Still, the high failure rate after a negative CTPA warrants ongoing vigilance.

## Introduction

COVID-19 disease ranges from a mild disorder with flulike symptoms to a critical care respiratory condition requiring intensive care unit (ICU) admission and mechanical ventilation. 1 2 Patients with COVID-19 are known to be at high risk for thrombotic complications, especially (but not exclusively) when admitted to the ICU. The most frequent thrombotic complication is acute pulmonary embolism (PE). 3 4 5 6 7 8

Diagnosing PE is long recognized to be challenging, as signs and symptoms of PE—for instance shortness of breath, coughing, and chest pain—are nonspecific and show overlap with mimicking conditions, including respiratory tract infections. 9 Imaging tests are required to confirm or rule out the diagnosis, and as a consequence many patients are referred for diagnostic imaging, with a low proportion of confirmed cases among those tested. 10 These imaging tests are associated with radiation exposure and contrast material-induced complications. 11 12

Diagnosing PE in the setting of COVID-19 is particularly challenging as signs and symptoms of PE and COVID-19 overlap, D-dimer levels are often elevated in the absence of thrombosis, 1 2 and computed tomography pulmonary angiography (CTPA) may be unfeasible in the case of respiratory or hemodynamic instability or in patients requiring mechanical ventilation at the ICU. Moreover, as CTPA may show in situ immunothrombosis, 13 14 for which the optimal treatment is unknown, rather than acute thromboembolism, widespread use of CTPA as screening test may lead to treatment dilemmas and overtreatment.

Guidance on the best diagnostic approach for suspected PE in (suspected) COVID-19 patients is lacking. While diagnostic strategies, including clinical pretest probability assessment using validated clinical decision rules and D-dimer testing, are recommended in international guidelines, including consensus documents dealing with COVID-19, 15 16 its use has not been prospectively validated in the setting of COVID-19. We set out to evaluate safety and efficiency of validated diagnostic strategies for ruling out PE in patients with (suspected) COVID-19.

## Methods

### Study Design and Patients

In a prospective, multicenter, outcome study we included patients with both (suspected) COVID-19 and clinically suspected acute PE. COVID-19 was considered confirmed in case of a positive polymerase chain reaction (PCR) test or in patients with a negative PCR but highly suggestive symptoms and typical COVID-19 abnormalities on CT-scan of the chest (CO-RADS 4 or 5 following Dutch Radiology Society 17 ) in the absence of an alternative diagnosis. Patients were included between March 1 ^st^ , 2020 and October 29 ^th^ , 2020 in four university hospitals and 10 nonuniversity teaching hospitals across the Netherlands and one hospital in Dublin, Ireland. Diagnostic management of suspected PE was performed at the discretion of the treating physician, based on local protocols.

Outpatients and inpatients (both ward and ICU) with clinically suspected acute (first or recurrent) PE were eligible for inclusion if they were aged 18 years or older. At the discretion of the treating physician, PE was suspected in COVID-19 patients based on new onset or worsening of chest pain or dyspnea, new/unexplained tachycardia, a fall in blood pressure not attributable to tachyarrhythmia, hypovolemia, electrocardiogram changes suggestive of PE and increasing D-dimer levels over time. Exclusion criteria included treatment with therapeutic doses of anticoagulants initiated 24 hours or more before eligibility assessment. None of the participating hospitals followed a strategy with screening for either acute PE or deep vein thrombosis in COVID-19 patients at admission.

Informed consent for use of patient's data was obtained by an opt-out approach in all included patients. This study was approved by the Institutional Review Board of the LUMC for observational studies, a decision endorsed by all other Dutch study sites, and institutional approval was also granted at the study site in Dublin (Ireland), and was performed on behalf of the Dutch COVID & Thrombosis Coalition (DCTC). 18

### Procedures

The treating physician assessed the patient and ordered diagnostic testing for ruling out PE, based on local hospital protocols and clinical judgment. Patients were managed by validated diagnostic strategies for suspected PE, including YEARS 19 20 or Wells 21 22 , or immediately received CTPA without assessment of pretest probability (“CTPA only”). Patients in whom PE was ruled out at baseline did not receive therapeutic anticoagulation and were followed for 3 months. Follow-up consisted of a scheduled outpatient visit or telephone interview after 3 months. At this visit, information about incident suspected venous thromboembolism (VTE) during follow-up was obtained. Patients in whom acute PE was confirmed at baseline were treated with anticoagulants according to international guidelines, in absence of contraindications. Baseline characteristics and information on the applied diagnostic strategy and follow-up were collected using standardized electronic case report forms (eCRF).

The decision to perform CTPA in patients in whom the YEARS algorithm was followed was made after assessing the YEARS items and the D-dimer level. In the absence of any of the YEARS items and a D-dimer level of less than 1,000 ng/mL, PE was considered to be ruled out without CTPA. In patients with one or more of the three YEARS items and a D-dimer level of less than 500 ng/mL, PE was also considered to be ruled out without CTPA. All other patients were referred for CTPA to confirm or rule out the diagnosis of PE. 19 In patients managed according to the Wells rule, this rule was combined with D-dimer testing in patients with unlikely clinical pretest probability, using a fixed (500 ng/mL) or age-adjusted D-dimer threshold (age × 10 ng/mL for patients above 50 years). PE was considered excluded in patients with an unlikely clinical probability score and a normal D-dimer test. All other patients were referred for CTPA. 21 22 The last management strategy applied in our study was CTPA in all patients with suspected PE (“CTPA only”), independent from pretest probability or D-dimer test result.

### Outcomes

The primary outcome was the 3-month incidence of (imaging confirmed) symptomatic VTE in patients in whom the diagnosis of PE was ruled out at baseline, and in whom therapeutic anticoagulant treatment was withheld, also referred to as the diagnostic failure rate. The failure rate was calculated in patients managed with and without CTPA separately, for all strategies under study. The diagnosis of PE or deep-vein thrombosis (DVT) was based on results of imaging tests (CTPA/ventilation-perfusion scan [VQ] and compression ultrasonography [CUS], respectively), or based on a high clinical suspicion if imaging could not be performed (i.e., because of respiratory or hemodynamic instability). VTE outcomes were centrally adjudicated by two physicians, independent of each other. Deaths were classified as caused by PE if it was confirmed by autopsy, was shown by objective testing before death, or could not be confidently excluded as a cause of sudden death. For patients managed according to YEARS or Wells, the secondary outcome was the number of patients in whom CTPA was not indicated to rule out PE, also referred to as the efficiency of the diagnostic strategy.

### Statistical Analysis

Patient baseline characteristics and information on the applied diagnostic strategy for ruling out PE were described using standard descriptive statistics. The primary outcome, which assessed the safety of the diagnostic strategy, and the analysis of the secondary outcome, which assessed the efficiency of the diagnostic strategy, were reported as percentages with corresponding 95% confidence intervals. SPSS Statistics version 25.0 served for data analysis.

### Role of the Funding Source

This study was funded by unrestricted grants of the participating hospitals and the Dutch COVID & Thrombosis Coalition was funded by the Netherlands Thrombosis Foundation and The Netherlands Organization for Health Research and Development. The steering committee, consisting of the authors, had final responsibility for the study design, oversight, and data verification and analyses. The sponsor was not involved in the study. All members of the steering committee contributed to the interpretation of the results, approved the final version of the manuscript, and vouch for the accuracy and completeness of the data reported. The final decision to submit the manuscript was made by the corresponding author on behalf of all co-authors.

## Results

### Patients

From March 1, 2020, to October 29, 2020, 730 patients with (suspected) COVID-19 were suspected of acute PE in the 14 participating hospitals; 23 patients (3.2%) were excluded because they already received therapeutic anticoagulation therapy at baseline. As a result, 707 patients were included in this study.

Patient baseline characteristics are summarized in Table 1 . The mean age was 62 years (SD 15), 398 patients (56%) were male, and the median body mass index was 27 (interquartile range [IQR]: 24–30). In addition, 45 patients (6.4%) had a history of VTE, and 73 patients (10%) had concurrent active cancer. In 424/707 patients (60%) the diagnosis of COVID-19 was ultimately confirmed, either by a positive PCR test or based on highly suggestive symptoms with typical COVID-19 abnormalities on CT-scan of the chest and no alternative diagnosis. Although the other 283 patients (40%) were suspected for COVID-19 at the time of suspected PE event, this COVID-19 diagnosis could ultimately not be confirmed because PCR testing was negative or was not performed, or because the CT scan was avoided because of the applied PE diagnostic strategy. A total of 151 patients (21%) were admitted to the ICU at the moment of study inclusion. Overall, PE was detected at baseline in 197 patients (28%), of whom 151 patients were ultimately diagnosed with COVID-19 (77%) and in 46 patients COVID-19 diagnosis could ultimately not be confirmed (23%).

**Table 1:** Baseline characteristics

*Baseline characteristics*	*N* = 707
Age (mean, SD)	62 (15)
Male sex (number, %)	398 (56)
Body mass index (median, IQR)	27 (24–30)
Active cancer (number, %)	73 (10)
Prior history of VTE (number, %)	45 (6.4)
Pregnant (number, %)	8 (1.1)
Admitted to the ICU at the time of suspected PE event (number, %)	151 (21)
Ultimately confirmed a COVID-19 disease (number, %)	424 (60)

Abbreviations: ICU, intensive care unit; IQR, interquartile range; PE, pulmonary embolism; SD, standard deviation; VTE, venous thromboembolism.

a COVID-19 status was confirmed in patients with a positive polymerase chain reaction (PCR) test or considered positive in patients with a negative PCR but highly suggestive symptoms and typical COVID-19 abnormalities on CT-scan of the chest (CO-RADS 4 or 5 following Dutch Radiology Society) with no alternative diagnosis (testing was not always available at baseline yet, and sometimes confirmed afterward).

### Diagnostic Management

A total of 255 patients (36%) were managed according to the YEARS algorithm, 30 patients (4.2%) were managed according to the Wells rule, and 370 patients (52%) were managed with CTPA only. Fifty-two patients (7.4%) were not tested for PE due to hemodynamic or respiratory instability. CUS of the legs was performed in three of the latter, diagnosing DVT in two. Therapeutic anticoagulant therapy was started in 30 of the 50 patients in whom the presence of PE remained unclear (60%).

### YEARS Algorithm

**Fig. 1:**
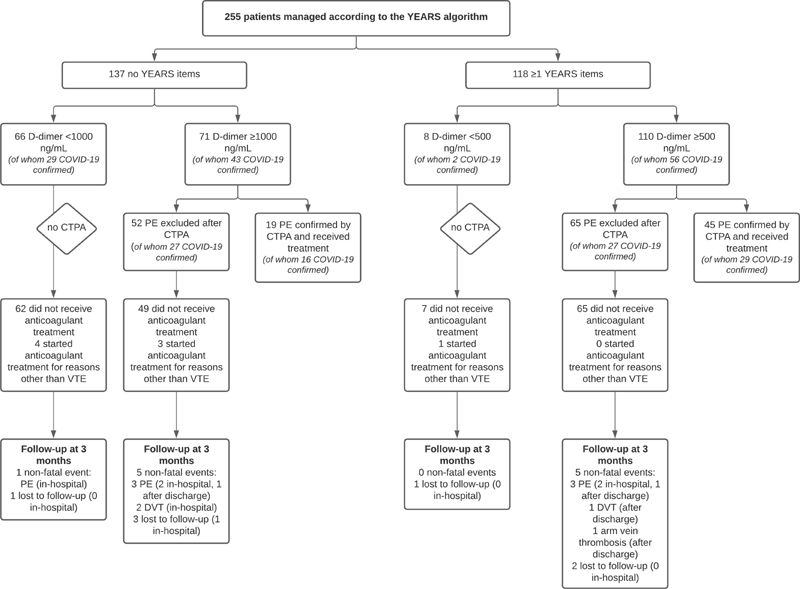
Flowchart of study patients managed according to the YEARS algorithm. CTPA, computed tomography pulmonary angiography; DVT, deep-vein thrombosis; PE, pulmonary embolism; VTE, venous thromboembolism.

Of the 255 patients managed by YEARS, 196 were admitted to the hospital (77%), 31 were admitted to the ICU at time of suspected PE event (12%), and 130 were ultimately diagnosed with COVID-19 (51%). In addition, 47 patients presented with fever (>38°C; 18%) and the median D-dimer level was 1,320 ng/mL (IQR 627–4,058 ng/mL). In total, 137 patients (54%) scored 0 YEARS items, 112 patients (44%) scored 1 YEARS item, and six patients (2.4%) scored 2 YEARS items. The item “PE most likely diagnosis” was scored most often (109/255 cases, 43%). In 74/255 patients (29%), PE was considered excluded without CTPA (66 patients with no YEARS items; eight patients with ≥1 YEARS items). Of those, five received anticoagulant therapy for other reasons than VTE. Among the 69 patients who remained untreated, one patient with confirmed COVID-19 was diagnosed with nonfatal PE during follow-up (failure rate 1.4%; 95% CI 0.04–7.8; Fig. 1 and Table 2 ) and two patients were lost to follow-up. Of the 117 patients with a negative CTPA, three patients received anticoagulant treatment for other reasons than VTE and one patient was lost to follow-up while still hospitalized (transferred to another hospital). Of the remaining 113 patients, 10 patients were diagnosed with nonfatal VTE (failure rate 8.8%; 95% CI 4.3–16; Table 3 ) and four were lost to follow-up after discharge from hospital. CTPA was positive and confirmed PE in 64 patients (19 patients 0 YEARS items, 45 patients ≥1 YEARS items; overall PE prevalence 25%). Therapeutic anticoagulant therapy was started in 63/64 patients, of whom none were diagnosed with recurrent VTE during follow-up.

**Table 2:** Diagnostic failures in patients who were managed with the YEARS algorithm—without CTPA—at baseline

	Sex	Age (years)	YEARS score	D-dimer concentration (ng/mL)	COVID-19 ultimately confirmed	Interval (days)	Outcome	Circumstances of outcome event	Adjudicated as
Patient 1	Male	77	0	970	Yes	2	Pulmonary embolism	Patient admitted to hospital (ward) at baseline. Dyspnea was already present since 2 wk. After 2 d of admission acute respiratory deterioration with elevated oxygen demand. CTPA scan was of moderate quality due to extensive ground glass consolidations. CTPA result: no central PE, suspicion of subsegmental PE in the right upper lobe.	Nonfatal subsegmental pulmonary embolism

Abbreviations: CTPA, computed tomography pulmonary angiography; PE, pulmonary embolism.

**Table 3:** Diagnostic failures in patients who were managed with the YEARS algorithm—after negative CTPA—at baseline

	Sex	Age (years)	YEARS score	D-dimer concentration (ng/mL)	COVID-19 ultimately confirmed	Interval (days)	Outcome	Circumstances of outcome event	Adjudicated as
Patient 1	Male	58	1	740	Yes	14	Arm vein thrombosis	Post-discharge patient received a CUS of the left arm because of pain symptoms. CUS was positive for arm vein thrombosis (thrombus in cephalic vein at the level of the elbow). Whether patient had a catheter in this arm during hospitalization was unknown.	Arm vein thrombosis
Patient 2	Female	53	0	5,900	Yes	55	Deep-vein thrombosis	Patient was intubated and admitted to the ICU. CUS was performed during hospitalization on the ICU because of suspected DVT of the right leg. CUS confirmed DVT at the level of the iliac vein and femoral vein. Patient had a catheter in situ for dialysis.	Deep-vein thrombosis
Patient 3	Male	76	1	1,315	No	32	Deep-vein thrombosis	Patient with a medical history of previous VTE and a heterozygote prothrombin mutation, visited post-discharge GP because of complaints of the left leg. CUS was positive for a DVT (at the level of popliteal vein until external iliac vein).	Deep-vein thrombosis
Patient 4	Male	72	0	1,160	No	9	Deep-vein thrombosis	Patient with a medical history of active malignancy, was hospitalized because of stem cell transplantation. Respiratory deterioration during hospitalization. Patient was admitted to the ICU and was clinically suspected of PE. CTPA was at that time not possible (due to hemodynamic instability) and CUS was performed by the intensivist at the ICU. CUS was positive for DVT (femoral vein). CTPA was still performed after 5 more days, which was negative for PE.	Deep-vein thrombosis
Patient 5	Female	33	1	796	No	3	Pulmonary embolism	Patient admitted to hospital (ward) at baseline. After 3 d of admission there was an acute deterioration with elevated oxygen demand. CTPA scan showed no central PE but confirmed subsegmental PE in the right lower lobe.	Nonfatal subsegmental pulmonary embolism
Patient 6	Male	56	0	1,546	No	12	Pulmonary embolism	Patient with a medical history of active malignancy. Was not admitted at baseline. Follow-up scan for malignancy revealed incidental PE (bilateral segmental).	Nonfatal pulmonary embolism
Patient 7	Female	43	1	800	No	36	Pulmonary embolism	Patient with a medical history of active malignancy. Post-discharge patient visited the ER because of lower back pain. CTPA was positive for PE (saddle embolus left pulmonary artery).	Nonfatal pulmonary embolism
Patient 8	Male	54	0	22,400	Yes	67	Pulmonary embolism	Patient was admitted to the ICU at baseline and intubated. Initial CTPA at baseline was negative for PE. Patient required high oxygen demands. Two weeks later patient developed a pneumothorax. 1-wk later patient was extubated and discharged to the ward. One week thereafter patient was readmitted to the ICU because of respiratory insufficiency (due to pneumonia and persistent pneumothorax). CTPA was negative for PE (performed on +36 d from baseline). Patient was re-intubated again. During re-admission on ICU a new CTPA scan was performed because of respiratory decline. CTPA report showed a contrast abnormality in the right lower lobe, suggesting subsegmental PE.	Nonfatal subsegmental pulmonary embolism
Patient 9	Male	54	1	5,000	Yes	5	Pulmonary embolism	Patient was intubated and admitted to the ICU. New CTPA was performed because of persistent elevated D-dimer values and clinical deterioration. CTPA revealed bilateral subsegmental PE.	Nonfatal subsegmental pulmonary embolism
Patient 10	Male	69	0	1,070	Yes	9	Pulmonary embolism	Patient was admitted to the hospital (ward). New CTPA was performed during hospitalization because of respiratory decline. CTPA was positive for bilateral segmental PE.	Nonfatal pulmonary embolism

Abbreviations: CTPA, computed tomography pulmonary angiography; CUS, compression ultrasonography; DVT, deep-vein thrombosis; ER, emergency department; GP, general practitioner; ICU, intensive care unit; PE, pulmonary embolism; VTE, venous thromboembolism.

### Wells Rule

The Wells rule plus either fixed or age-adjusted D-dimer threshold was applied in only 30 patients, of whom one patient was admitted to the ICU (3.3%) and nine were ultimately diagnosed with COVID-19 (30%). Two out of 30 patients could be managed without CTPA (6.7%). Twenty-three patients had a negative CTPA (77%) and remained untreated, of whom one patient developed DVT (failure rate 4.3%, 95% CI 0.11–22; Table 4 ) and eight were lost to follow-up. PE was confirmed with CTPA in five patients (17%), all received therapeutic anticoagulant therapy, and none developed recurrent VTE during follow-up.

**Table 4:** Diagnostic failures in patients who were managed with the Wells rule—after negative CTPA—at baseline

	*Sex*	Age (years)	COVID-19 ultimately confirmed	Interval (days)	Outcome	Circumstances of outcome event	Adjudicated as
Patient 1	Male	41	No	8	Deep-vein thrombosis	Patient with a medical history of active malignancy. During hospitalization swollen right light and thus suspected DVT. CUS confirmed DVT (right leg: at the level of femoral vein).	Deep-vein thrombosis

Abbreviations: COVID-19, coronavirus disease 2019; CUS, compression ultrasonography; DVT, deep-vein thrombosis.

### Directly Imaged with CTPA (“CTPA Only”)

**Fig. 2:**
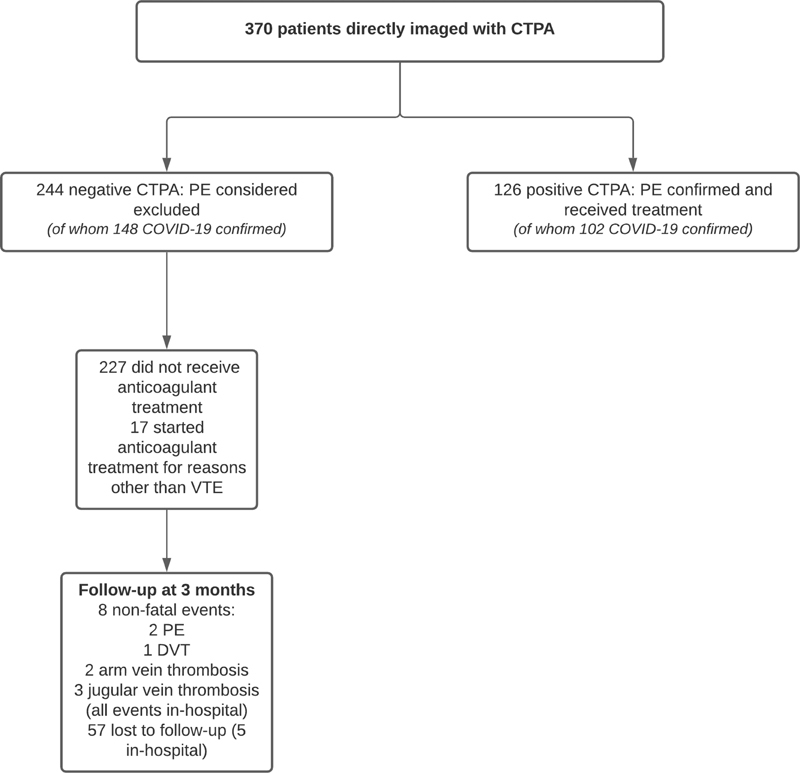
Flowchart of study patients directly imaged with CTPA. CTPA, computed tomography pulmonary angiography; DVT, deep-vein thrombosis; PE, pulmonary embolism; VTE, venous thromboembolism.

CTPA was directly performed in 370 patients (52%). Of these 370 patients, 340 were admitted to the hospital (92%), 101 were admitted to the ICU at the time of suspected PE event (27%), and 250 were ultimately diagnosed with COVID-19 (68%). In addition, 122 patients presented with fever (>38°C; 33%). Of the 370 patients, 244 had a negative CTPA ruling out PE at baseline (66%), of whom 17 received therapeutic anticoagulation for other reasons than VTE and five were lost to follow-up while still hospitalized (transferred to another hospital). Among the 222 patients in whom PE was ruled out and who remained untreated during follow-up, eight patients were diagnosed with nonfatal VTE (failure rate 3.6%; 95% CI 1.6–7.0; Fig. 2 and Table 5 ); 52 were lost to follow-up after discharge from hospital. CTPA confirmed PE in the other 126 patients (overall prevalence PE 34%), of whom 120 received therapeutic anticoagulant therapy and five were subsequently diagnosed with recurrent VTE during follow-up.

**Table 5:** Diagnostic failures in patients who were managed with “CTPA alone”—after negative CTPA—at baseline

	Sex	Age (years)	COVID-19 ultimately confirmed	Interval (days)	Outcome	Circumstances of outcome event	Adjudicated as
Patient 1	Male	63	Yes	11	Jugular vein thrombosis	Patient was intubated and admitted to the ICU. Central catheter was noticed to not work well anymore. CUS was performed by the intensivist at the ICU, which revealed thrombus surrounding the central catheter (bilateral).	Jugular vein thrombosis
Patient 2	Male	57	Yes	6	Jugular vein thrombosis	Patient was intubated and admitted to the ICU. CUS was performed of the left jugular vein (reason unknown), by the intensivist at the ICU. CUS confirmed jugular vein thrombosis (surrounding central catheter).	Jugular vein thrombosis
Patient 3	Male	20	Yes	6	Arm vein thrombosis	Patient with a history of active malignancy. CUS was performed during hospitalization because of a swollen right arm. CUS revealed a small thrombus, surrounding central catheter (vein itself not occluded), in the right axillar vein.	Arm vein thrombosis (catheter tip)
Patient 4	Male	53	Yes	4	Arm vein thrombosis	Patient was intubated and admitted to the ICU. Thrombosis of the left arm was suspected during ultrasound-guided IV injection. CUS confirmed arm vein thrombosis later on (basilar vein).	Arm vein thrombosis
Patient 5	Male	50	Yes	8	Pulmonary embolism	Patient was admitted to the ward at baseline. 1 d later patient was transferred to the ICU and intubated because of respiratory deterioration. PE was clinically suspected because of increase in ventilated to perfused lung areas, but CTPA could not be performed due to the clinical condition of the patient. Some days later CTPA could be performed and confirmed the PE diagnosis (bilateral segmental and subsegmental PE).	Nonfatal pulmonary embolism
Patient 6	Female	71	Yes	3	Pulmonary embolism	Patient was admitted to the ward at baseline. Two days later patient was transferred to the ICU and intubated because of respiratory deterioration. One day later PE was clinically suspected but CTPA could not be performed because of hemodynamic instability. Heparin was started because of high respiratory demands, highly elevated D-dimer values, and a medical history of active metastasized malignancy (breast cancer). Six days later it was decided by thrombosis specialists that CTPA would not be beneficial anymore, since a negative CTPA would not rule out PE from a couple of days ago.	Clinically suspected nonfatal pulmonary embolism (not radiologically confirmed)
Patient 7	Female	46	No	15	Deep-vein thrombosis	Patient was immediately admitted to the ICU at baseline, because of respiratory insufficiency. PE and/or COVID-19 was suspected, but diagnosis was ultimately not confirmed after further testing. Final diagnosis was decompensation cordis in the presence of endocarditis and patient underwent mitral valve replacement. Patient underwent CT with contrast material because of peritonitis some days later; scan revealed thrombus in the inferior vena cava (at the top of central catheter in the inguinal area).	Deep-vein thrombosis
Patient 8	Male	52	Yes	16	Jugular vein thrombosis	Patient was intubated and admitted to the ICU. CUS of the right jugular vein was performed during hospitalization, and revealed jugular vein thrombosis (catheter related).	Jugular vein thrombosis

Abbreviations: CTPA, computed tomography pulmonary angiography; CUS, compression ultrasonography; DVT, deep-vein thrombosis; ICU, intensive care unit; PE, pulmonary embolism; VTE, venous thromboembolism.

## Discussion

An important unanswered question in the clinical arena of COVID-19 is the optimal diagnostic approach of suspected acute PE. Results of our prospective study underline the applicability of the YEARS algorithm, as CTPA could be avoided in 29% of patients managed by YEARS, with a low failure rate. Importantly, the failure rate of a negative CTPA (within YEARS or used as a sole test) reflects the high thrombotic risk in these patients and emphasizes the importance of remaining alert for incident (new) VTE during follow-up.

Up to now, diagnostic strategies for suspected PE have not been prospectively validated in patients with COVID-19, and only small retrospective studies on this topic have been published. 23 24 25 As elevated D-dimer levels are common in COVID-19 patients, strategies using a fixed D-dimer threshold of 500 ng/mL have limited ability to exclude PE without CTPA, as was demonstrated in a study applying the Wells rule with a fixed D-dimer threshold wherein only 2% of patients had a negative D-dimer. 23 Our study shows that, with the use of the YEARS algorithm, 19 CTPA could be avoided in 29% of patients (74/255), at a low diagnostic failure rate (1.4%; 95% CI 0.04–7.8). Importantly, while the upper limit of the 95% CI has turned out higher due to the relatively small number of patients included in this analysis, the point estimate is acceptably low. Moreover, this failure rate was also lower than in the patients who did receive CTPA (within YEARS or CTPA used as a sole test: failure rate 8.8% and 3.6%, respectively). Using the Wells rule, CTPA was avoided in only two patients (6.7%) and 23/30 patients had a negative CTPA (77%; failure rate 4.3% 95% CI 0.11–22). Despite performing computed tomography in nearly all (hospitalized) COVID-19 patients (to determine CT severity score), avoidance of CTPA and contrast material is warranted given the potential complications, as for instance contrast-induced nephropathy. The threshold of 1,000 ng/m for D-dimer using YEARS is likely to be beneficial in patients with COVID-19, since a considerable number of COVID-19 patients—varying between 18 and 53%—in previous studies had D-dimer values below 1,000 ng/mL, 26 27 28 29 but only 2 to 26% below 500 ng/mL. 23 27 30

Another observation deserves comment for clinical practice in this COVID-19 setting. The failure rate of a negative CTPA, used as a sole test (3.6%) or within YEARS (8.8%) or Wells (4.3%), was considerably higher than reported for other (non-COVID-19) patients with suspected PE, where failure rates mostly vary between 1 and 3%. 19 31 In our study, most of these “diagnostic failures” were observed while patients with COVID-19 were still hospitalized, and despite pharmacological thromboprophylaxis. This higher failure rate is to be expected in patients with a high PE risk, as is dictated by Bayes' theorem. 10 COVID-19 patients who are hospitalized are at increased risk for developing VTE, and importantly, remain at risk after initial negative testing for developing new (de novo) thrombotic events during follow-up. Of note, the failure rate of a negative CTPA within YEARS was higher than the failure rate of a negative CTPA used as a sole test (8.8% vs. 3.6%, respectively). This is explained by the fact that patients receiving CTPA within YEARS were preselected to be at high risk for PE based on clinical parameters and D-dimer level.

Our study has strengths and limitations. The major strength of this study is the prospective multicenter study design by which we prospectively evaluate diagnostic strategies for suspected PE in the setting of COVID-19. Other strengths include the large sample size and the detailed data collection using a standardized protocol and eCRF. An important limitation is that YEARS was not implemented as standard diagnostic strategy across all participating hospitals. Subsequently, patients with severe COVID-19 illness were more often managed with the “CTPA only” strategy, which is supported by the findings in our study, as patients in the “CTPA only” strategy were more frequent admitted to the ICU. Still, this real-world setting adds to the value and generalizability of our findings. Furthermore, as results of COVID-19 testing were not always available at baseline, patients with suspected COVID-19 were also eligible for inclusion. Therefore, not all patients included in this study had ultimately confirmed COVID-19 disease. However, it is important to recall that—because of the shortage in PCR COVID-19 tests in the first wave—patients who presented to the emergency department (ER) and did not require admission to the hospital were often not tested. As a consequence, COVID-19 diagnosis was neither confirmed nor completely rejected in these patients. Regardless of this point, it was not possible to perform subgroup analyses for patients with confirmed COVID-19 alone, due to the small sample size in the different study arms. Nowadays, rapid diagnostic testing for COVID-19 is widely available and diagnostic uncertainty is therefore reduced to a minimum. Yet, we believe that the results of this study are still applicable to today's patients, since half of the patients managed by YEARS had confirmed COVID-19 disease and only one diagnostic failure was observed—during hospital admission—in patients not receiving imaging. These results support the use of diagnostic strategies in patients with suspected PE, also in the setting of COVID-19. Another limitation of this study was that one suspected PE event during follow-up could not be imaging confirmed, because CTPA was impossible due to hemodynamic instability. After adjudication this event was nevertheless added as a diagnostic failure. Importantly, we choose to calculate the failure rate based on all confirmed VTE events during follow-up. This included also arm vein thrombosis, jugular vein thrombosis, and catheter tip thrombosis, despite the fact that it is unlikely that these VTE events indeed represent a missed PE diagnosis at baseline. This approach has led to a very conservative and higher observed failure rate. We nevertheless considered it important to give this overall picture of these thrombotic episodes of our (suspected) COVID-19 study population.

In conclusion, our results underline the applicability of the YEARS algorithm in COVID-19 patients with suspected PE in view of the avoidance of CTPA in 29% of patients at an acceptably low failure rate. The high failure rate of a negative CTPA points to the need of remaining vigilant for new incident VTE during follow-up, and the relevance of a low threshold for ordering new diagnostic tests, should the clinical situation deteriorate.

## Notes

**What Is Known about This Topic?**

- Hospitalized patients with COVID-19 are at high risk for thrombotic complications, the most frequent thrombotic complication observed is pulmonary embolism.
- Diagnosing PE in the setting of COVID-19 is particularly challenging as signs and symptoms overlap, D-dimer levels are often elevated in the absence of thrombosis and CTPA may be unfeasible in the case of respiratory or hemodynamic instability.
- Diagnostic strategies for suspected PE have not been prospectively validated in the setting of COVID-19.

**What Does This Paper Add?**

- In our prospective study, CTPA could be avoided in 29% of patients by using the YEARS algorithm, at an acceptable low diagnostic failure rate (1.4%), which underlines the applicability of YEARS in this setting.
- Importantly, the failure rate after a negative CTPA (within YEARS or used as a sole test) was non-negligible, reflecting the high thrombotic risks in these patients and emphasizing the importance of remaining alert for new incident VTE during follow-up.

## Notes

Appendix A The Dutch COVID and Thrombosis Coalition Study Group Authors' Contribution M.A.M.S. gathered and verified data, performed the analyses, and primarily drafted the first version of the manuscript. F.H.J.K., R.H.H.B., T.v.B., I.C.B., D.C.W.B., S.J.E.B, C.B., H.t.C., D.D.D., L.M.F., M.J.J.H.G., L.R.d.H., C.H., A.I.d.S., S.K., T.K., R.I.M., F.P., E.R.E.v.T., P.E.W., and M.t.W. gathered data and revised the manuscript critically for important intellectual content. L.J.M.K. revised the manuscript critically for important intellectual content. F.A.K. designed the study, performed the analyses, and drafted the first version of the manuscript and revised the manuscript critically for important intellectual content. M.V.H. designed the study, verified data, performed the analyses, and drafted the first version of the manuscript and revised the manuscript critically for important intellectual content. All authors agree with the final version.
