# Supplementary material for: Ruling out Pulmonary Embolism in Patients with (Suspected) COVID-19—A Prospective Cohort Study
Source: TH Open. 2021 Sep 15;5(3):e387–99. doi: 10.1055/s-0041-1735155 (PMC8443402; doi:10.1055/s-0041-1735155)
Supplement: Supplementary file 1 — Supplementary Appendix B [file 10-1055-s-0041-1735155-s210051.pdf]

Consortium members

Amsterdam University Medical Center:

- Location AMC:

Prof. Dr. D. van de Beek, neurologist
Dr. M. C. Brouwer, neurologist
Drs S. de Bruin, phd-candidate
Dr. M. Coppens, vascular medicine specialist
Drs. N. van Es, phd candidate
Drs. T. F. van Haaps, phd-candidate Department of vascular medicine
Prof. dr. N. P. Juffermans, intensivist
Prof. dr. S. Middeldorp, vascular medicine specialist
Dr. M. C. A. Muller, intensivist
Dr. A. P. J. Vlaar, intensivist

- Location VUMC:

Prof. dr. C. M. P. M. Hertogh, nursing home specialist
Prof. Dr. L. M. A. Heunks, professor intensive care
Drs. J. G. Hugtenburg, pharmacologist
Dr. J. van Kooten, nursing home specialist
Dr. E. J. Nossent, pulmonologist
Prof. Dr. Y. Smulders, internist
Dr. P. R. Tuinman, intensivist
Dr. A. Vonk Noordegraaf, pulmonologist

Amphia Hospital:
Dr. M. J. J. H Grootenboers, pulmonologist
Dr. C van Guldener, internist
Dr. M. Kant, pulmonologist

Argos zorggroep:
Drs. A. Lansbergen, physiotherapist

Erasmus Medical Center:
Drs. J. van den Akker, intensivist
Dr. R. Bierings, cellular biologist, Department Haematology
Dr. H. Endeman, intensivist.
Dr. M. Goeijenbier, internal medicine, Department Viroscience
Dr. N. G. M. Hunfeld, hospital pharmacist
Prof. dr. E. C. M. van Gorp, infectious diseases specialist, Department Viroscience
Prof. dr. D. A. M. P. J. Gommers, intensivist
Prof. dr. M.P.G. Koopmans, internist, Department Viroscience.
Prof. dr. T. Kuiken, professor of comparative pathology, Department Viroscience
Drs. T. Langerak, phd-candidate, Department Viroscience
Dr. M. N. Lauw, haematologist, Department Haematology
Prof. dr. M. P. M. de Maat, head of Biochemistry of Haemostasis and Thrombosis.
Drs. D. Noack, phd-candidate i.o. Department Viroscience
Drs. M.S. Paats, pulmonologist
Drs. M.P. Raadsen, phd-candidate Department Viroscience
Dr. B. Rockx, internist, Department Viroscience
Dr. C. Rokx, infectious diseases specialist
Dr. C. A. M. Schurink, infectious diseases specialist
Drs. K. Tong-Minh, phd-candidate Department Viroscience
Dr. L. van den Toorn, pulmonologist
Dr. C. A. den Uil, Cardiologist-Intensivist
Drs. C. Visser, phd-candidate, Department haematology

Farmadam:

Drs. F. Boutkourt, phd-candidate
Drs. T. Roest, pharmacist

Flevo Hospital:
Dr. R. A. Douma, infectious diseases specialist
Drs. L. R. de Haan, phd-candidate
Dr. M. ten Wolde, vascular medicine specialist

Hospital Group Gelderse Vallei:
Dr. R. H. H. Bemelmans, internist
Dr. B. Festen, intensivist

Ikazia Hospital:
Dr. S. Stads, intensivist

Jeroen Bosch Hospital:
Dr. C. P. C. de Jager, intensivist
Dr. K.S. Simons, intensivist

Leiden University Medical Center:
Drs. M.L. Antoni, cardiologist, Department of cardiology
Dr. M. H. Bos, biochemicus, associate professor, Department of Medicine - Thrombosis and Hemostasis
Drs. J. L. I, Burggraaf, phd-candidate , Department of Clinical Epidemiology
Prof. S. C. Cannegieter, clinical
epidemiologist, Department of Medicine - Thrombosis and Hemostasis and Department of Department of Clinic
al Epidemiology
Prof. dr. H. C. J. Eikenboom, haematologist/vascular medicine specialist
, Department of Medicine - Thrombosis and Hemostasis.
Dr. P. L. den Exter, vascular medicine specialis, Department of Medicine - Thrombosis and Hemostasis
Dr. J. J. M. Geelhoed, pulmonologist, Department of Pulmonology
Prof. dr. M. V. Huisman, vascular medicine specialist, Department of Medicine - Thrombosis and Hemostasis
Prof. E. de Jonge, internist-intensivist, Department of Intensive Care Medicine
Drs. F. H. J. Kaptein, phd-candidate, Department of Medicine - Thrombosis and Hemostasis
Dr. F. A. Klok, vascular medicine specialist, Department of Medicine - Thrombosis and Hemostasis
Dr. L. J. M. Kroft, radioloog, Department of radiology
Drs. L. Nab, phd-candidate, Department of Clinical Epidemiology
Dr. M. K. Ninaber, pulmonologist, Department of Pulmonology
Prof. dr. H. Putter, statistician, Department of Biomedical Data Sciences
Drs. S. R. S. Ramai, pulmonologist, Department of Pulmonology
Dr. A. M. da Rocha Rondon, Postdoctoral researcher, Department of Medicine - Thrombosis and Hemostasis
Dr. A. H. E. Roukens, infectious diseases specialist, Department of infectious diseases
Drs. M. A. M. Stals, phd-candidate, Department of Medicine - Thrombosis and Hemostasis
Prof. dr. H .H. Versteeg, cellular biologist, Department of Medicine - Thrombosis and Hemostasis
Dr. H. W. Vliegen, cardiologist, Department of cardiology
Dr. B. J. M. van Vlijmen, cellular
biologist, associate professor, Department of Medicine - Thrombosis and Hemostasis

Maastricht University Medical Center:
Drs. T. van de Berg, phd-candidate
Drs. R. Bruggemann, phd-candidate
Dr. B. C. T. van Bussel, internist-intensivist
Prof. dr. H. ten Cate, Internist
Dr. A. Ten Cate-Hoek, Clincial epidemiologist and medical director of thrombosis service Maastricht
Prof. dr. T. M. Hackeng, biochemist
Dr. ir. Y. Henskens, clinical chemist
Drs. A. Hulshof, phd-candidate
Drs. M. Mulder, phd-candidate

Prof. dr. L. Schurgers, biochemist
Dr. B. Spaetgens, internist subspecialised in geriatrics
Dr. H. Spronk, biochemist
Prof. dr. M. A. Spruit, executive board member Ciro and Professor in Rehabilitation
Dr. K. Winckers, vascular medicine specialist

Maxima Medical Center:
Dr. L. Nieuwenhuizen, haematologist

Medical Center Leeuwaarden:
Drs. B. Franken, haematologist
Dr. I. M. Schrover, vascular medicine specialist
Drs. E. G. M. de Waal, haematologist

Medical Center Twente:
Dr. A. Beishuizen, intensivist

Radboud University Medical Center:
Dr. J. Leentjens, vascular medicine specialist
Dr. Q. de Mast, infectious diseases specialist
Prof. dr. K. Kramer, professor medical safety

Reinier de Graaf Gasthuis hospital:
Dr. R. E. Brouwer, haematologist
Dr. J. L. J. Ellerbroek, infectious diseases specialist
Drs. J. Tijmensen, haematologist

Rijnstate hospital:
Dr. M. M. C. Hovens, vascular medicine specialist
Dr. E. A. N. Oostdijk, intensivist
Drs. B. D. Westerhof, Anaesthesiologist-intensivist

Rode Kruis Hospital:
Dr. L. M. Faber, Haematologist

Sanquin Research, Amsterdam:
Dr. M. van den Biggelaar, head of Laboratory of Proteomics, Department of Molecular and Cellular Hemostasis
Prof. dr. J.C.M. Meijers, biochemist (and Amsterdam University Medical Centers)
Prof. dr. J. Voorberg, molecular en cellular biologist (and Amsterdam University Medical Centers)

St Fransiscus Gasthuis & Vlietland Hospital:
Dr. M. E. Kevenaar, internist
Drs. Y. L. Soei, internist
Dr. E. J. Wils, intensivist

St. Jansdal Hospital:
Dr. F. N. Croles, haematologist

Synapse Research Institute:
Dr. B. de Laat, biochemist, director

Tergooi hospital:
Prof. Dr. P. W. Kamphuisen, vascular medicine specialist
Dr. R. Vink, intensivist

University Medical Center Groningen:
Prof. dr. T. Lisman, biochemist

Prof. dr. K. Meijer, haematologist, Department Haematology
Dr. Y. I. G. van Tichelaar, internist

University Medical Center Utrecht:
O.L. Cremer, anesthesiologist-intensivist
Dr. G. Geersing, general practicioner, Julius Center, Department primary care
Prof dr. H.A.H. Kaasjager, vascular medicine specialist
Dr. N. Kusadasi, haematologist-intensivist
Drs. A. D. Huisman, dietician
Dr. M. Nijkeuter, vascular medicine specialist
Prof. dr. R.E.G. Schutgens, haematologist, Van Creveldkliniek
Dr. R.T. Urbanus, biochemist, Van Creveldkliniek
Drs. J. Westerink, vascular medicine specialist

Wilhelmina Hospital Assen:
Dr. H. J. Faber, internist-intensivist

Zaans Medical Center:
Drs. S. Koster, anesthesiologist-intensivist

Zuyderland Hospital:
Dr. P. van Montfort, resident internal medicine
Dr. D. J. L. van Twist, vascular medicine specialist
